# Presynaptic GABA Receptors Mediate Temporal Contrast Enhancement in *Drosophila* Olfactory Sensory Neurons and Modulate Odor-Driven Behavioral Kinetics

**DOI:** 10.1523/ENEURO.0080-16.2016

**Published:** 2016-08-23

**Authors:** Davide Raccuglia, Li Yan McCurdy, Mahmut Demir, Srinivas Gorur-Shandilya, Michael Kunst, Thierry Emonet, Michael N. Nitabach

**Affiliations:** 1 Department of Cellular and Molecular Physiology, Yale University School of Medicine, New Haven, Connecticut 06520; 2 Interdepartmental Neuroscience Program, Yale University, New Haven, Connecticut 06520; 3 Department of Molecular, Cellular and Developmental Biology, Yale University, New Haven, Connecticut 06520; 4 Department of Physics, Yale University, New Haven, Connecticut 06520; 5 Department of Genetics, Yale University School of Medicine, New Haven, Connecticut 06520; 6 Program in Cellular Neuroscience, Neurodegeneration and Repair, Yale University School of Medicine, New Haven, Connecticut 06520

## Abstract

Contrast enhancement mediated by lateral inhibition within the nervous system enhances the detection of salient features of visual and auditory stimuli, such as spatial and temporal edges. However, it remains unclear how mechanisms for temporal contrast enhancement in the olfactory system can enhance the detection of odor plume edges during navigation. To address this question, we delivered to *Drosophila melanogaster* flies pulses of high odor intensity that induce sustained peripheral responses in olfactory sensory neurons (OSNs). We use optical electrophysiology to directly measure electrical responses in presynaptic terminals and demonstrate that sustained peripheral responses are temporally sharpened by the combined activity of two types of inhibitory GABA receptors to generate contrast-enhanced voltage responses in central OSN axon terminals. Furthermore, we show how these GABA receptors modulate the time course of innate behavioral responses after odor pulse termination, demonstrating an important role for temporal contrast enhancement in odor-guided navigation.

## Visual Abstract

**Figure:**
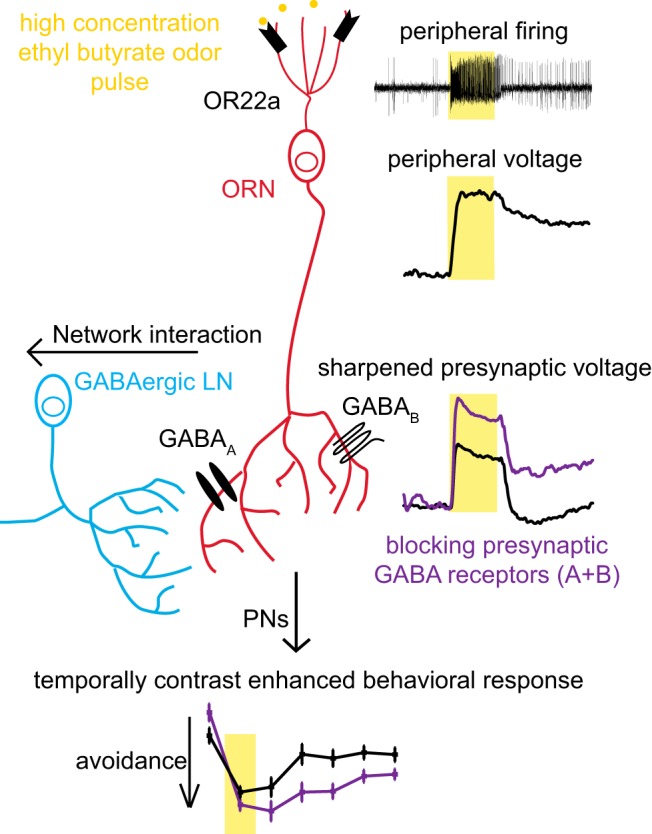

## Significance Statement

Contrast enhancement of visual, auditory, and olfactory information shapes the spatial and temporal perception of our environment. The cellular mechanisms that mediate temporal contrast enhancement of olfactory information and their impact on behavior are not fully understood. We therefore use optical electrophysiology to investigate how presynaptic GABA receptors in olfactory sensory neurons of *Drosophila melanogaster* shape olfactory information and how this affects odor-driven behavioral kinetics. We find that the combined activity of two types of inhibitory GABA receptors mediates temporal contrast enhancement and modulates behavioral kinetics after an odor pulse, demonstrating the importance of this mechanism for odor-guided navigation.

## Introduction

Incoming sensory stimuli trigger network activity involving mutually connected excitatory and inhibitory neurons. Integration of these opposing signals is essential for robust environmental perception (Anderson et al., 2000; Wehr and Zador, 2003; Poo and Isaacson, 2009). In the mammalian retina, for example, lateral inhibition mediated by GABAergic interneurons enhances contrast sensitivity, and, thus, the ability to discriminate spatial differences in light intensities underlying object and motion detection (Kuffler, 1953; Buldyrev and Taylor, 2013). Animals also rely on the sense of smell to navigate their environments (Wallace et al., 2002). Odor plumes emitted by food sources and distributed by air currents guide sniffing and are temporally encoded by olfactory sensory neurons (OSNs) enabling odor-directed navigation (Mylne and Mason, 1991; Cury and Uchida, 2010; Shusterman et al., 2011; Celani et al., 2014). How inhibitory GABA receptors modulate neuronal activity to mediate temporal contrast enhancement and how it affects odor-driven behavioral kinetics is not fully understood.

In the vertebrate olfactory bulb (OB), GABAergic local interneurons (LNs) provide presynaptic inhibition to OSNs (Lledo et al., 2004). Presynaptic inhibition of OSNs mediates gain control, maintenance, and refinement of odor-specific neuronal activity within and between glomeruli (Urban, 2002; McGann et al., 2005; Pírez and Wachowiak, 2008). While it is clear that presynaptic inhibition maintains the encoding of odor identity over a wide range of odor intensities, it is unknown whether presynaptic inhibition also mediates temporal contrast enhancement. The anatomies of the mammalian and *Drosophila* olfactory systems are remarkably similar (Ache and Young, 2005; Kaupp, 2010). In *Drosophila*, the dendrites and cell bodies of OSNs reside in sensilla on the antennae and maxillary palps (Vosshall et al., 1999; Vosshall et al., 2000; Couto et al., 2005). Presynaptic OSN axon terminals expressing the same olfactory receptor protein converge onto glomeruli in the antennal lobe (AL), a neuropil analogous to the mammalian OB (Vosshall et al., 1999; Vosshall et al., 2000; Couto et al., 2005). Presynaptic inhibition by *Drosophila* LNs is known to mediate gain control (Olsen and Wilson, 2008; Root et al., 2008) and the refinement of odor-evoked spatial patterns of activation of glomeruli (Silbering and Galizia, 2007). More recently, postsynaptic electrical recordings have been used to show that presynaptic inhibition enables broadband transmission of rapidly fluctuating odor pulses and sharpens responses to brief transient stimuli (Nagel et al., 2015). Due to technical restraints, it was thus far not possible to directly visualize the effects of presynaptic inhibition on electrical responses in presynaptic terminals. Moreover, it remains unclear how presynaptic GABA receptors affect postpulse neuronal activity directly in the OSN terminals and how this activity can affect odor-guided navigation with respect to temporal contrast enhancement.

We therefore used voltage imaging to directly compare electrical activity in the peripheral somata of OSN with their presynaptic terminals. Interestingly, we found that these odor stimuli induce sustained postpulse responses in the peripheral somata and dendrites of OSNs. The activity of presynaptic ionotropic GABA_A_ and metabotropic GABA_B_ inhibitory receptors generates contrast-enhanced voltage responses in OSN terminals, and also accelerates behavioral responses to the termination of an intense odor pulse. We demonstrate how presynaptic GABA receptors modulate neuronal activity to mediate gain control and temporal contrast enhancement, which together improve behavioral performance and could enhance plume-guided navigation.

## Materials and Methods

### Experimental preparation

Fly stocks were raised on standard cornmeal food at 25°C and 60% humidity under a 12 h light/dark regime. Wild-type *Drosophila* strains Canton-S and w1118 were used in this study. The following transgenic lines were used: *UAS-ArcLight* (Cao et al., 2013); *UAS-GCaMP6f* (Chen et al., 2013); *UAS-pdf-RNAi* (Ni et al., 2009); *UAS-GABA_A_-RNAi* (8-10G, used for all physiological experiments and 2-7E2; Liu et al., 2009); *UAS-GABA_B_-RNAi* (Root et al., 2008); *UAS-Dcr-2* (Dietzl et al., 2007); and *Or22a-GAL4*, *Or42b-GAL4, OR42a-GAL4* (Vosshall et al., 2000).

For recordings of olfactory responses, female flies between 3 and 7 d posteclosion were used. For imaging and electrophysiological recordings of the antennae, standard procedures were used (de Bruyne et al., 2001). For immobilization, a fly was pushed all the way into a truncated 200 µl pipette tip. One of the exposed antennae was then stabilized between a tapered glass micropipette and double-sided tape attached to a cover glass. For imaging of the central brain, the preparation was modified after the method of Fiala and Spall (2003). Briefly, flies were anesthetized and, using two-component adhesive epoxy, immobilized on sticky tape attached to a hole-punched plastic coverslip. To further immobilize the head, an insect pin was gently pushed against the head and attached to the plastic coverslip using Paraplast wax. After the cuticle was exposed, a thin layer of epoxy was used to seal gaps between the head and the sticky tape. After letting the epoxy harden for 30–60 min, the cuticle above the head, air sacks, and glands were removed under insect saline containing the following (in mm): NaCl 103, KCl 5, CaCl_2_ 2, MgCl_2_ 4, NaHCO_3_ 26, NaH_2_PO_4_ 1, TES 5, trehalose 10, and glucose 10, pH 7.4. Picrotoxin (PTX; Abcam) and CGP54626 (CGP; Tocris Bioscience) were dissolved as 20 mm stock solution in DMSO, and then diluted in insect saline and used as 200 and 100 µm.

### Imaging and electrophysiology

Imaging was performed on a Zeiss Axio Examiner upright microscope using a Plan Apochromat 40×, numerical aperture (NA) 1.0, water-immersion objective (Zeiss, Germany) for imaging of the central brain, and a LMPlanFl 50×, NA 0.5, air-objective (Olympus) for imaging of the antenna. Using a Colibri LED system (Zeiss), ArcLight and GCamp were excited at 470 nm. LED power was adjusted for each recording individually to make sure that fluorescent image was not saturated. The objective C-mount image was projected onto the 80 × 80 pixel chip of a NeuroCCD-SM camera controlled by NeuroPlex software (RedShirtImaging). For image demagniﬁcation, we used an Optem C-to-C mount 25-70-54 0.38× (LINOS, Qioptiq). Voltage imaging was performed at a frame rate of 125 Hz, and calcium imaging was performed at a frame rate of 40 Hz. Optical traces were obtained as spatial averages of intensity of all pixels within the region of interest, with signals processed as reported elsewhere (Jin et al., 2012; Cao et al., 2013) with double-exponential ﬁtting to compensate for rapid and slow photobleaching followed by eight rounds of box-car smoothing.

Single sensillum recordings (SSRs) were performed as described previously (de Bruyne et al., 2001). Electrical signals were amplified using an Iso-DAM amplifier (World Precision Instruments), bandpass filtered (300 Hz to 2 kHz), digitized at 10 kHz (NI-USB6221 Digital Acquisition Board, National Instruments), and acquired using data acquisition software that is freely available at https://github.com/sg-s/kontroller. Spikes were identified and sorted using a spike-sorting toolbox available at https://github.com/sg-s/spikesort.

### Odor delivery

**Figure 1.**
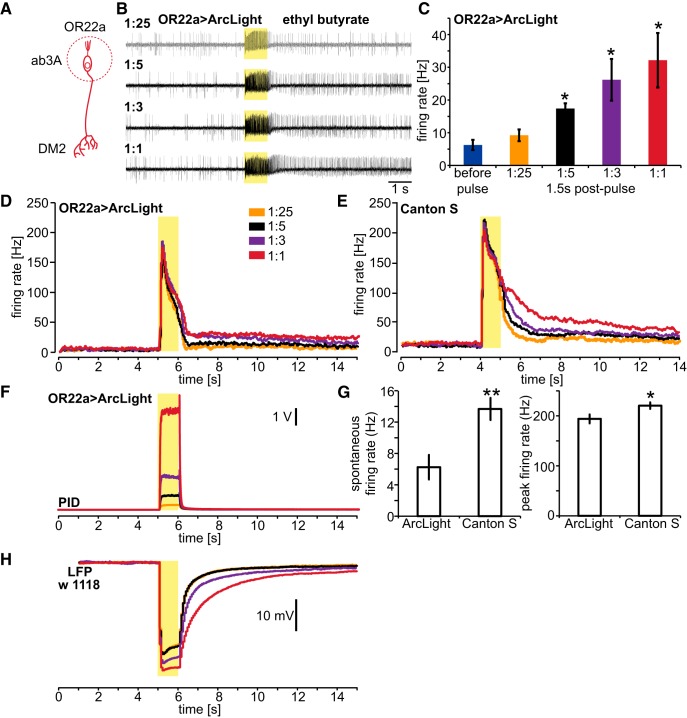
Increasing odor concentrations induces sustained peripheral OSN responses. ***A***, An OSN depicting the dendrites and cell body in the antenna and the presynaptic axon terminals in the antennal lobe. The circle indicates focus on odor-elicited activity in the dendrites and cell body. ***B***, Extracellular SSRs of action potentials in an ab3 sensillum, which contains OR22a-expressing OSNs. One second pulses of Eb of the indicated gas-phase dilutions were delivered during the indicated interval (yellow box). Recordings are representative of those obtained from four flies and 10 sensilla per concentration. ***C***, Comparison between the spontaneous and postpulse firing rate at 1.5 s after odor pulse offset shows sustained firing for odor intensities of ≥1:5. Mean ± SEM; *n* = 4. Statistical analysis: one-way ANOVA for repeated measurements with Dunn’s *post hoc* test (spontaneous firing as control); *p < 0.05. ***D***, Mean firing rates (*n* = 4 flies) of a neuron recorded in ab3 sensilla of ArcLight-expressing flies. ***E***, Mean firing rates (*n* = 4 flies) of a neuron recorded in ab3 sensilla of Canton S flies. ***F***, Representative odor signals measured at the outlet of the odor delivery system using a PID. ***G***, OR22a-OSNs expressing ArcLight display reduced spontaneous and odor-induced peak firing rates compared with wild-type OR22a-OSNs (Canton S). Data from ***D*** and ***E*** were averaged across concentrations. Statistical analysis: unpaired *t* test, *p < 0.05; **p < 0.01. ***H***, Mean LFP (*n* = 3) of ab3 sensilla in w1118 flies showing sustained neuronal activity.

Odorants were delivered using a custom gas-phase dilution olfactometer. Compressed medical air (Airgas) was split into three airstreams, and the flow rate of each airstream was regulated with mass flow controllers (Alicat Scientific). Two airstreams were combined to create the specific odor dilutions. One of those two airstreams passed over a 20 ml glass vial containing 5 ml of pure ethyl butyrate (Eb; 99%; Sigma-Aldrich). This airstream was combined with the other airstream, which passed through an empty vial. By changing the ratios of flow rates between the two airstreams, various gas-phase dilutions of the odorant were obtained. A computer-controlled three-way solenoid valve (NResearch Inc.) delivered the odorized airstream either to a waste outlet or to the fly via a glass delivery tube ∼2 cm away from the fly. For all imaging experiments, the total air volume directed at the fly was 600 ml/min. The odor concentration was monitored using a photo ionization detector (200B, Aurora Scientific), which was placed either directly adjacent to the fly (2–4 mm) or at the opening of the odor delivery tube. For all purely electrophysiological experiments (Fig. 1), we used two mass flow controllers to dilute odorants in a secondary airstream with a flow rate of 200 ml/min. This secondary airstream was diverted into a main airstream (2000 ml/min) using computer-controlled solenoid valves (Lee Co.). The photoionization detector (PID) was used to make sure that dilutions using this odor delivery system were comparable to the dilutions used during imaging experiments.

### Innate avoidance and attraction assay

For behavioral experiments, we used a custom-built arena, comprising a circular acrylic base (10 cm diameter) and a Petri dish lid that enclosed the arena. Four openings for odor ports were drilled into the outer layer of the circular base (each 90°). The height inside the arena was 1.5 mm, providing sufficient height for the flies to walk but not to fly. The arena was illuminated from the bottom by an LED light box (Huion). Videos were collected at 30 frames per second (fps) using a high speed digital camera (Casio EX-FC150).

Experiments were conducted in a dark room maintained at 50% humidity. To increase locomotor activity, experiments were conducted at 30°C (Zaninovich et al., 2013; Clark et al., 2014). The 3- to 7-d-old flies were food deprived for 12–24 h in vials with wet Kimwipes. Before each experiment, 30–50 male and female flies were allowed to acclimatize inside the arena for 5 min. Odor pulses were subsequently presented in increasing concentrations with interpulse intervals of 1 min. The odor port used was randomized for each experiment from among four available in the arena. For 10 s pulse experiments, each concentration was tested twice. For 1 s pulse experiments, each concentration was tested once. After each experiment, flies were discarded, and the arena and odor tube were aired for 10 min to clear residual odorant before performing the next experiment.

The detection and tracking of the flies were performed using a modified open-source MATLAB code (http://studentdavestutorials.weebly.com/kalman-filter-with-matlab-code.html). For 10 s pulse experiments, the location of each fly and its distance from the odor port was calculated every 15 frames (0.5 s) for a total of 35 s, and the average distance from odor port over time was plotted. For 1 s pulse experiments, flies were individually tracked at 30 fps over a period of 4.5 s, and the relative distance moved with respect to the initial position of each fly during odor onset (Δ*d*) was calculated. We defined Δ*d_t_* as the difference in distance from the odor port between odor onset and time *t* (Δ*d_t_* = *d*_0_ − *d_t_*), such that positive Δ*d_t_* values reflect movement toward the odor port (i.e, attraction) and negative values reflect movement away (i.e., avoidance).

## Results

### Sustained peripheral OSN responses measured by classic and optical electrophysiology

**Figure 2.**
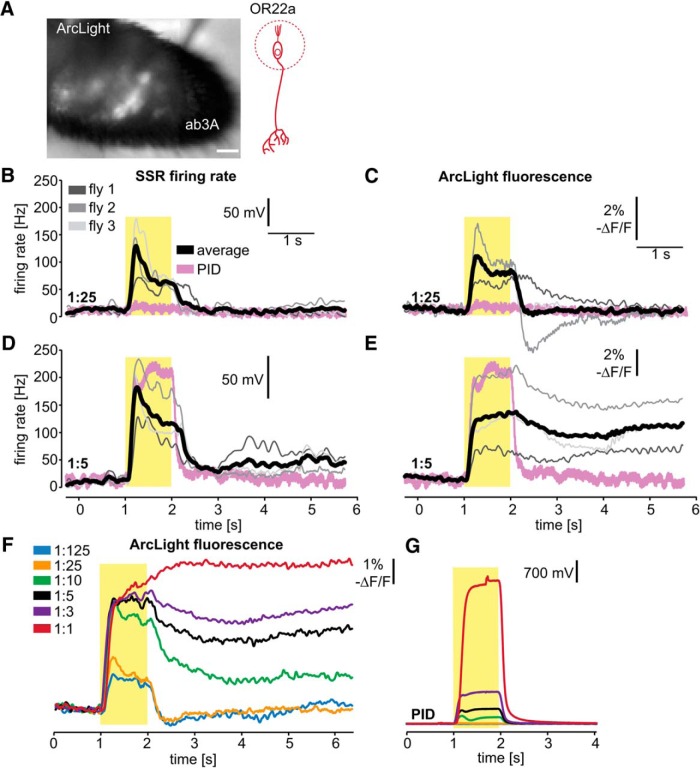
Optical electrophysiology reveals sustained peripheral OSN responses. ***A***, Combined fluorescent and transmitted light image of the antenna of a fly expressing ArcLight in OR22a-expressing OSNs. Scale bars, 20 µm. ***B–E***, Simultaneous SSR of ab3 and voltage imaging of the antenna of three flies expressing ArcLight in OR22a-expressing OSNs. Representative PID signals are shown and were measured ∼2–4 mm behind the fly. Yellow boxes indicate odor pulse duration. ***F***, Mean ArcLight signals (*n* = 4) in response to 1 s Eb pulses at the indicated gas-phase dilutions. ***G***, Mean PID signals for the odor pulses in ***F***, measured at the fly.

As previously reported, increasing odor concentrations tend to prolong peripheral firing in OSNs (Martelli et al., 2013). To investigate whether this prolonged firing is subject to central processes of contrast enhancement, we first determined odor concentrations that induce sustained peripheral odor responses. We chose the fruit-typical odor Eb, which strongly activates a large number of olfactory receptors (Hallem and Carlson, 2006). Because Eb elicits the strongest response in OR22a-expressing OSNs (de Bruyne et al., 2001), we measured the peripheral responses of these neurons to Eb pulses (Figs. 1, 2). We used both extracellular SSRs (Fig. 1) and high-speed imaging of the ArcLight genetically encoded fluorescent voltage indicator expressed in OR22a-expressing OSNs (Fig. 2). The SSR of OR22a-expressing OSNs in the ab3 sensillum indicates that Eb pulses at gas-phase dilutions of 1:5 and higher odor concentrations elicit sustained post-pulse action potential firing trains (Fig. 1*A–D*, for statistics see Table 1). To determine the intensity and time course of odor delivery at the different gas-phase dilutions, we directly measured odor intensity with a PID positioned at the odor outlet (Fig. 1*F*) as in the study by Martelli et al. (2013). To exclude the possibility that the expression of ArcLight alters OSN excitability to induce these sustained responses, we performed SSR on ab3 sensilla of wild-type Canton S flies, revealing that they also exhibit sustained responses after the termination of Eb pulses (Fig. 1*E*). However, compared with Canton S, the expression of ArcLight leads to reduced spontaneous and odor-induced peak firing rate in OR22a-expressing OSNs (Fig. 1*G*), indicating that ArcLight reduces neuronal excitability. This is most likely because ArcLight adds capacitance to the neurons, as shown for pigment dispersing factor (PDF)-expressing neurons (Cao et al., 2013). As ArcLight does not increase excitability, the observed sustained neuronal activity cannot be due to the expression of ArcLight. We also measured the local field potential (LFP) for another strain of flies (LFP of w1118; Fig. 1*H*), which also indicates sustained neuronal activity. Whether the sustained peripheral activity is due to postpulse lingering odor or intracellular cascades triggered at high concentrations remains to be investigated. We further chose to focus on whether the observed sustained activity is subject to central processes of contrast enhancement modulating odor-driven behavioral kinetics.

**Table 1:** Statistical evaluation

Figure	Initial statistical test	*Post hoc* test
1*C*	Kruskal–Wallis one-way ANOVA, *H* = 21.05,*p* < 0.001	Dunn; before pulse vs 1:25: Q = 0.73, *p* > 0.05; before pulse vs 1:5, 1:3, 1:1: Q > 2.5, *p* < 0.05
1*G*	Unpaired *t* test, *t* = −3.27, *p* = 0.006 for spontaneous firing rate and *t* = -2.21, *p* = 0.049 for peak firing rate	
3*E*	Two-way ANOVA, *F* = 6.15, *p* = 0.003	Bonferroni vs peripheral voltage; at 1:125, 1:25, 1:10: vs presynaptic voltage and presynaptic Ca^2+^: *t* < 1.96, *p* > 0.09;at 1:5, 1:1: vs presynaptic Ca^2+^: *t* < 1.47, *p* > 0.29; vs presynaptic voltage: *t* > 3.11, *p* < 0.005
3*I*	Two-way ANOVA, *F* = 7.63, *p* = 0.008	Bonferroni; at 1:25: *t* = 0.55, *p* = 0.59; at 1:5: *t* = 2.09, *p* = 0.04; at 1:3: *t* = 2.24, *p* = 0.03; at 1:1: *t* = 0.84, *p* = 0.41
3*N*	Two-way ANOVA, *F* = 268.5, *p* < 0.001	Bonferroni; at 1:125: *t* = 0.07, *p* = 0.95; at 1:25: *t* = 0.49, *p* = 0.63; at 1:5: *t* = 14.85, *p* < 0.001; at 1:1: *t* = 17.37, *p* = 0<0.001
4*G*	Two-way ANOVA for repeated measurements, *F* = 42.46, *p* < 0.001	Bonferroni; for CGP: *t* = 0.55, *p* = 0.59; for PTX *t* = 1.28, *p* = 0.21; for CGP+PTX 1:5: *t* = 4.38, *p* < 0.001; for CGP+PTX 1:3:*t* = 8.59, *p* < 0.001; CGP+PTX 1:5 vs 1:3, *t* = 0.27, *p* = 1
4*H*	Two-way ANOVA for repeated measurements, *F* = 20.61, *p* < 0.001	Bonferroni; for CGP: *t* = 0.03, *p* = 0.98; for PTX *t* = 1.69, *p* = 0.1; for CGP+PTX 1:5: *t* = 3.87, *p* < 0.001; for CGP+PTX 1:3:*t* = 4.65, *p* < 0.001; CGP+PTX 1:5 vs 1:3, *t* = 3.42, *p* = 0.008
4*I*	Paired *t* test, *t* = -3.61, *p* = 0.011 for maximum response and *t* = 2.45, *p* = 0,049 for sharpness	
5*A*	Two-way ANOVA for repeated measurements, *F* = 32.7, *p* < 0.001	Bonferroni vs control; vs GABA_B_-RNAi: *t* > 2.81, *p* < 0.02; vs GABA_A_-RNAi: *t* = 1.72, *p* = 0.26 at 1:25, otherwise *t* > 3.05,*p* < 0.008; vs GABA_B_ + GABA_A_-RNAi: *t* > 2.93, *p* < 0.012
5*B*	Two-way ANOVA for repeated measurements, *F* = 9.91, *p* < 0.001	Bonferroni vs control; vs GABA_B_-RNAi: *t* < 1.07, *p* > 0.86; vs GABA_A_-RNAi: *t* < 1.13, *p* > 0.78; vs GABA_B_ + GABA_A_-RNAi:*t* = 0.15-0.18, *p* = 1 at 1:125 and 1:25; *t* = 2.49, *p* = 0.04 at 1:5; *t* = 3.15, *p* = 0.006 at 1:3; *t* = 5.12, *p* < 0.001 at 1:1
5*D*, *F*, *H*	Two-way ANOVA for repeated measurements, *F* > 326.31, *p* < 0.001	Bonferroni, control vs GABA_A_, values given for significant time intervals indicated in figure; at 1:25 *t* > 3.18, *p* < 0.004; at 1:5*t* > 3.09, *p* < 0.006; at 1:3, *t* > 2.64, *p* < 0.025
5*D*	Two-way ANOVA for repeated measurements, *F* > 326.31, *p* < 0.001	Bonferroni, control vs GABA_B_, values given for significant time intervals indicated in figure; at 1:25 *t* > 2.52, *p* < 0.035
5*F*, *H*, *J*	Two-way ANOVA for repeated measurements, *F* > 326.31, *p* < 0.001	Bonferroni, control vs GABA_A_ + GABA_B_ values given for significant time intervals indicated in figure; at 1:5 *t* > 2.56, *p* < 0.03; at 1:3*t* > 3.09, *p* < 0.006; at 1:1 *t* > 2.74, *p* < 0.018
6*G*	Two-way ANOVA, *F* = 26.7, *p* < 0.001	Bonferroni vs control; vs GABA_A_-RNAi, at 1:25 and 1:5 *t* = 3.56 and *t* = 3.59, *p* = 0.001; vs GABA_A_ + GABA_B_-RNAi (+Dicer), at 1:25:*t* = 4.72, *p* < 0.001 (*t* = 5.11, *p* < 0.001); at 1:5: *t* = 2.53, *p* = 0.046 (*t* = 5.32, *p* < 0.001); at 1:1: *t* = 2.83, *p* < 0.001 (*t* = 5.35, *p* < 0.001)
6*H*	Two-way ANOVA, *F* = 21.7, *p* < 0.001	Bonferroni vs control; vs GABA_A_-RNAi (27E2), at 1:25, *t* = 3.39 and*t* = 5.74, *p* < 0.001; at 1:1 *t* = 4.51 and *t* = 5.31, *p* < 0.001
7*A*	Two-way ANOVA, *F* = 7.17, *p* < 0.001	Bonferroni; differences between distances only significant for 1:1: 0-4 cm vs 4-10 cm, *t* > 2.98, *p* < 0.03
8*C*	Two-way ANOVA, *F* = 12.94, *p* < 0.001	Bonferroni vs control; at 0-1 post-pulse time *t* = 3.49-3.52, *p* = 0.009-0.002; at 2-3 s post-pulse time, *t* = 3.15-3.18, *p* = 0.007; at 3-4 s post-pulse time, *t* = 3.02, *p* = 0.01
8*D*	Two-way ANOVA, *F* = 6.63, *p* < 0.001	Bonferroni vs control; at 1-2 s post-pulse time, *t* = 3.33-3.6, *p* = 0.003-0.001; at 2-3 s post pulse time, *t* = 2.52-2.9, *p* = 0.047-0.015; at 3-4 s post pulse time, *t* = 3.29, *p* = 0.004

To test whether ArcLight-mediated optical electrophysiology can be used reliably to detect sustained peripheral electrical responses, we performed simultaneous SSRs from an ab3 sensillum and voltage imaging of the OR22a-expressing OSNs across the entire antenna (Fig. 2*B–E*). While SSRs can capture the firing rate of a single type of neuron, voltage imaging of the entire antenna averages the neuronal activity of all OR22a-expressing OSNs (Fig. 2*A*). To determine the time course of the odor stimulus experienced by the fly, we positioned the PID next to the fly (as is the case for all subsequent experiments). At a 1:25 dilution of Eb, the odor was barely detectable by the PID at the position of the fly (Fig. 2*B*). The dynamics of neuronal activity reported by ArcLight fluorescence (Fig. 2*C*) accurately recapitulates instantaneous firing frequency measured by SSR (Fig. 2*B*) and is in accordance with known features of OSN activation, including adaptation and poststimulus inhibition (Hallem and Carlson, 2006; Nagel and Wilson, 2011; Martelli et al., 2013). At the higher concentration (1:5 dilution), the odor pulse was readily detectable by the PID, and the sustained postpulse firing that lasts for several seconds becomes evident (Fig. 2*D*). Interestingly, at this high odor intensity the peripheral firing rate (Fig. 2*D*) and peripheral ArcLight fluorescence (Fig. 2*E*) are not identical. First, while sensory adaptation is pronounced in the firing rate, it is not present in the ArcLight fluorescence. Second, the firing rate shows a steep decline after offset and remains at ~20–40% of the offset firing rate (Fig. 2*D*). Arclight fluorescence shows no steep decline after offset and remains at ∼80–90% of the offset fluorescence signal (Fig. 2*E*). A possible explanation for these differences is that the ArcLight fluorescence also reflects the receptor potential, while the firing rate does not. In addition, it is also possible that, due to the dynamic range of ArcLight, the change in fluorescence during the odor pulse is saturated, and thus the differences in neuronal activity during and after the odor pulse are less pronounced. This would also mean that lower firing frequencies are represented more effectively by ArcLight compared with higher firing frequencies. However, we show that voltage imaging using ArcLight can readily detect postpulse sustained neuronal activity induced by increasing odor concentrations (Fig. 2*F*,*G*). The sustained peripheral response to such stimuli raises questions of whether, and how, the fly accurately detects the edge of an odor plume as it exits, a stimulus feature that is essential for accurate navigation (van Breugel and Dickinson, 2014).

### Temporal sharpening of odor-evoked voltage responses in OSN presynaptic terminals

**Figure 3.**
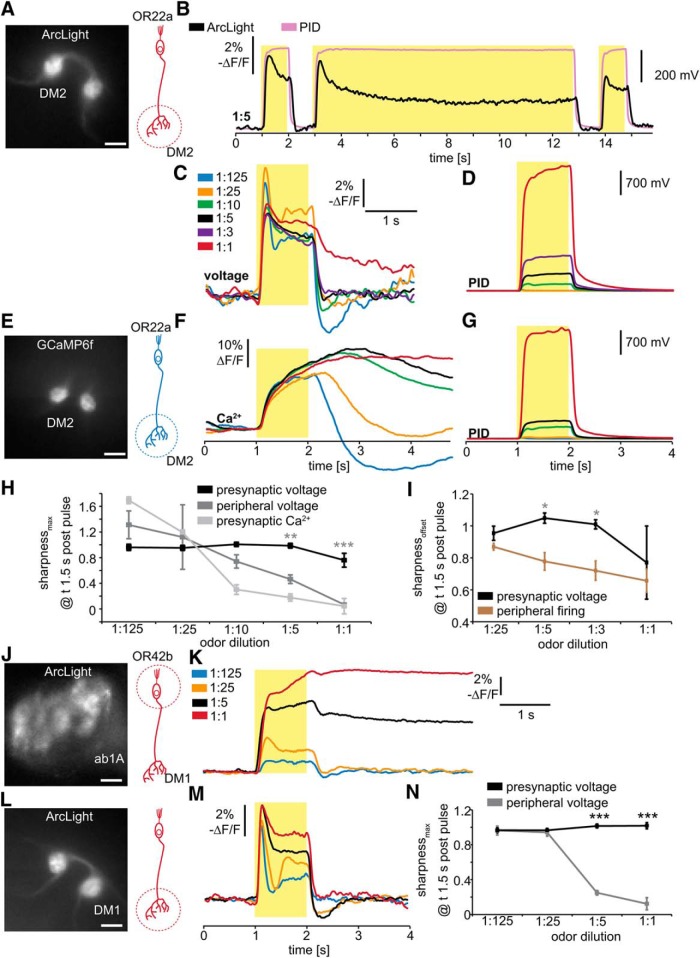
Optical electrophysiology of presynaptic axon terminals of OSNs indicates temporal contrast enhancement. ***A***, Fluorescent image of the antennal lobe of a fly expressing ArcLight in OR22a-expressing OSNs. Scale bars, 10 µm. The axon terminals of these neurons innervate the DM2 glomerulus. ***B***, Single-trial optical recording of presynaptic membrane potential in DM2 in response to pulses of 1:5 Eb, measured with the PID at the fly. Yellow boxes indicate odor pulse duration. ***C***, Mean presynaptic electrical responses (*n* = 5–11) in DM2 to 1 s Eb pulses of the indicated gas-phase dilutions. ***D***, Mean PID signals for the odor pulses in ***C***, measured at the fly. **E**, Fluorescent image of the antennal lobe of a fly expressing GCaMP6F in OR22a-expressing OSNs. Scale bar, 10 µm. ***F***, Mean presynaptic Ca^2+^ responses (*n* = 4–5) in DM2 to 1 s Eb pulses of the gas-phase dilutions indicated in ***C***. ***G***, Mean PID signals for the odor pulses in ***F***, measured at the fly. ***H***, Sharpness coefficient based on peak amplitude [(Δ*F*/*F*)_Max_ − (Δ*F*/*F*)_1.5 s postpulse_]/(Δ*F*/*F*)_Max_ of OSN voltage and Ca^2+^ responses in the antenna and AL. Sharpness of antennal voltage and presynaptic Ca^2+^ responses decreases with increasing odor concentration, while presynaptic voltage responses remain sharp, indicating the existence of a mechanism for temporal contrast enhancement of presynaptic electrical responses. Mean ± SEM; *n* = 4 for antennal voltage, *n* = 4-5 for presynaptic Ca^2+^, and *n* = 5-11 for presynaptic voltage. Statistical analysis: two-way ANOVA with Bonferroni *post hoc* test (antennal voltage as control); **p < 0.01; ***p < 0.001. ***I***, Sharpness coefficient based on neuronal activity at odor offset [(Δ*F*/*F* or Hz)_offset_ − (Δ*F*/*F* or Hz)_1.5 s postpulse_]/(Δ*F*/*F* or Hz)_offset_ comparing presynaptic voltage and peripheral firing rate of OSNs also indicates presynaptic contrast enhancement. Statistical analysis: two-way ANOVA with Bonferroni *post hoc* test; *p < 0.05. ***J***, Fluorescent image of the antenna of a fly expressing ArcLight in OR42b-expressing OSNs. Scale bars, 10 µm. ***K***, Mean peripheral electrical responses (*n* = 4) in OR42b-expressing neurons to 1 s Eb pulses of the indicated gas-phase dilutions. ***L***, Fluorescent image of the antennal lobe of a fly expressing ArcLight in OR42b-expressing OSNs. Scale bar, 10 µm. The axon terminals of these neurons innervate the DM1 glomerulus. ***M***, Mean presynaptic electrical responses (*n* = 6) in DM1 to 1 s Eb pulses of the indicated gas-phase dilutions. ***N***, Sharpness coefficient of peripheral voltage is reduced at high odor concentrations (1:5, 1:1), while presynaptic voltage responses remain sharp. Statistical analysis: two-way ANOVA with Bonferroni *post hoc* test, ***p < 0.001.

All OR22a-expressing OSN axons converge on the DM2 glomerulus in the AL, where their presynaptic terminals provide input to DM2-specific projection neurons (PNs; Couto et al., 2005). To determine the odor-induced synaptic inputs provided to these PNs, we directly measured odor responses of the presynaptic terminals of the OR22a-expressing OSNs with ArcLight imaging (Fig. 3*A–C*). In contrast to the sustained peripheral responses of OSNs upon termination of high-intensity Eb pulses (Figs. 1, 2), OSN presynaptic responses in DM2 decline rapidly back to baseline (Fig. 3*A–D*). In order to compare the kinetics of DM2 presynaptic membrane electrical responses with the kinetics of intracellular presynaptic Ca^2+^, we used GCaMP6f, the fastest available genetically encoded Ca^2+^ indicator (GECI; Chen et al., 2013; Fig. 3*E*). At the lowest odor intensity (1:125), the kinetics of presynaptic Ca^2+^ are similar to the kinetics of presynaptic voltage, exhibiting a temporally restricted increase and poststimulus inhibition (Fig. 3*F*,*G*). However, at higher odor concentrations, Ca^2+^ responses are dramatically sustained compared with the sharp electrical responses. This difference is likely explained by the fact that ArcLight measures electrical activity of the presynaptic membrane, while GECIs measure the bulk accumulation of presynaptic intracellular Ca^2+^.

In order to quantify temporal contrast enhancement we used a sharpness coefficient (sharpness_Max_), defined as [(Δ*F*/*F*)_Max_ − (Δ*F*/*F*)_1.5 s postpulse_]/(Δ*F*/*F*)_Max_. This formula represents the relative difference of neuronal activity between a time point during stimulation and afterward (1.5 s). A sharpness coefficient of 1 represents 100% temporal contrast of neuronal activity, while 0 represents no temporal contrast. The sharpness coefficient of peripheral electrical responses decreases with increasing odor intensity (Fig. 3*H*). In contrast, presynaptic electrical responses remain equally sharp across the entire range of tested odor intensities and are significantly sharper than peripheral responses to 1:1 and 1:5 Eb pulses (Fig. 3*H*). However, a direct comparison between peripheral and presynaptic ArcLight signal is complicated by the possibility that the peripheral ArcLight signal may reflect firing frequency and receptor potential, while spikes may travel more effectively to the presynaptic terminals than slower changes in membrane potential. We therefore directly compare the sharpness of the peripheral firing rate with the presynaptic ArcLight signal (Fig. 3*I*). To account for the pronounced sensory adaptation, we now use a sharpness coefficient that is based on the neuronal activity at odor offset (sharpness_offset_: (Δ*F*/*F*)_offset_ − (Δ*F*/*F*)_1.5 s postpulse_]/(Δ*F*/*F*)_offset_). This shows that presynaptic electrical responses to 1:5 and 1:3 dilutions are significantly sharper than the peripheral firing rate (Fig. 3*I*), which indicates that the sustained peripheral responses observed in Or22a-expressing neurons are temporally sharpened in their presynaptic terminals in the AL. At 1:1 sustained neuronal activity is also measurable in presynaptic terminals which could indicate that temporal sharpening can only be achieved up to a specific odor intensity. To test whether sharpened presynaptic voltage responses at high odor concentrations are found in other glomeruli, we measured OR42b-expressing OSNs, whose terminals project to the DM1 glomerulus (Fig. 3*J–N*). While peripheral voltage responses show sustained neuronal activity at odor dilutions of 1:5 and 1:1 (Fig. 3*K*), presynaptic voltage responses are significantly sharpened at these concentrations (Fig. 3*M*,*N*). The observed presynaptic sharpening could be a general mechanism for temporal contrast enhancement to improve edge detection and plume-guided navigation. To study this putative mechanism of temporal contrast enhancement, we further focused on optical electrophysiology as well as pharmacological and genetic manipulations to directly visualize presynaptic sharpening in the presynaptic terminals.

### Temporal contrast enhancement is mediated by presynaptic GABA receptors

**Figure 4.**
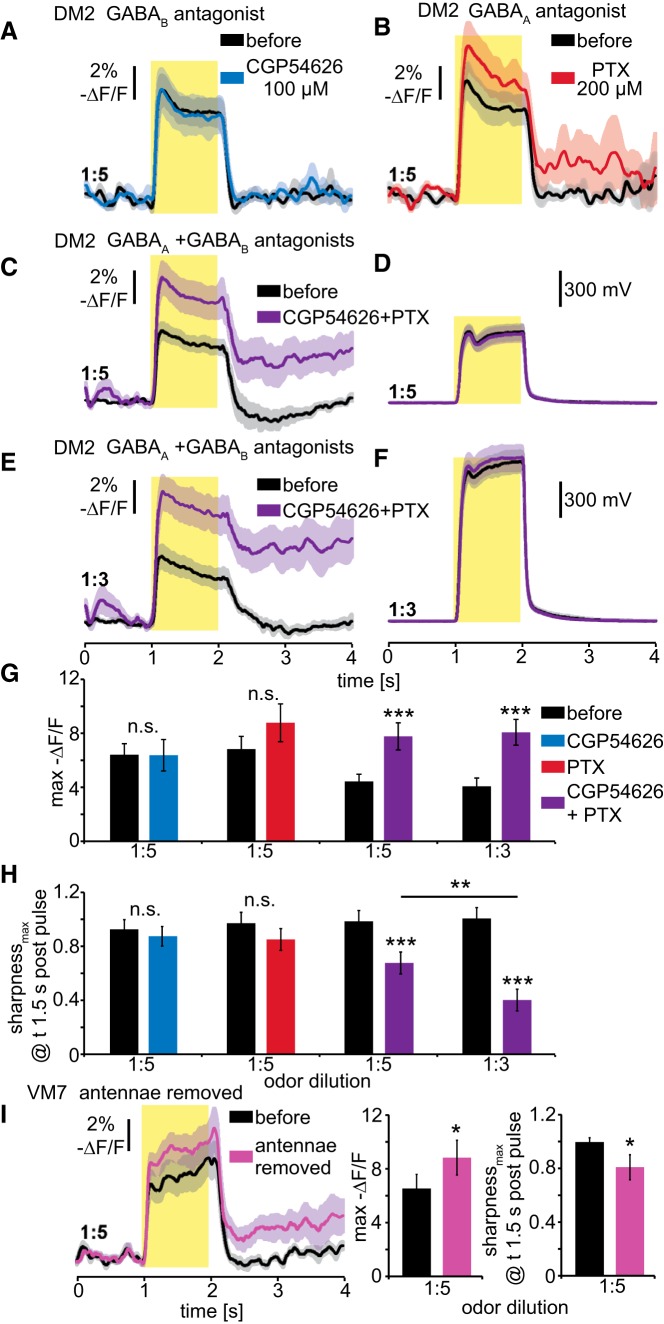
Temporal contrast enhancement in OSN presynaptic terminals is mediated by GABA_A_ and GABA_B_ receptors. ***A***, Pharmacological inhibition of GABA_B_ receptors with CGP54626 has no effect on presynaptic electrical responses of OR22a-expressing OSNs in DM2 to a 1 s pulse of 1:5 Eb. Mean ± SEM; *n* = 5. ***B***, Pharmacological inhibition of GABA_A_ receptors with PTX appears to slightly increase magnitude and prolong presynaptic electrical responses in DM2 to 1 s pulses of 1:5 Eb. Mean ± SEM; *n* = 5. ***C–F***, Simultaneous pharmacological inhibition of GABA_A_ and GABA_B_ receptors increases magnitude and prolongs presynaptic electrical responses in DM2 to 1 s pulses of 1:5 (***C***) and 1:3 (***E***) Eb. Simultaneously recorded PID signals are identical before and after drug application (***D***, ***F***). Mean ± SEM; *n* = 9. ***G***, Maximum presynaptic voltage responses indicate that only simultaneous pharmacological inhibition of GABA_A_ and GABA_B_ receptors significantly increases the magnitude of voltage responses. Mean ± SEM; *n* = 5 for CGP54626, *n* = 5 for PTX, and *n* = 9 for CGP54626+PTX. Statistical analysis: two-way repeated-measures ANOVA with Bonferroni *post hoc* test, ***p < 0.001. ***H***, Sharpness coefficient indicates that only simultaneous inhibition of GABA_A_ and GABA_B_ receptors significantly reduces the temporal contrast enhancement of presynaptic voltage responses. The sharpness of responses to 1:3 Eb is reduced significantly more than that to 1:5 Eb, which is consistent with the larger sustained peripheral response to 1:3 Eb (Fig. 2*F*). Mean ± SEM; *n* = 5 for CGP54626, *n* = 5 for PTX and *n* = 9 for CGP54626+PTX. Statistical analysis: two-way repeated-measures ANOVA with Bonferroni *post hoc* test, **p < 0.01; ***p < 0.001. ***I***, Removal of the antennae reduces lateral inhibition of the maxillary palp glomerulus VM7 (Olsen and Wilson, 2008). In response to 1:5 Eb, removal of the antennae increases presynaptic voltage responses and reduces sharpness. Mean ± SEM; *n* = 7. Statistical analysis: paired *t* test, *p < 0.05.

It has recently been shown that presynaptic inhibition of *Drosophila* OSNs promotes broadband synaptic transmission of olfactory stimuli by overcoming frequency restrictions imposed by short-term depression (Nagel et al., 2015). Moreover, this study shows that presynaptic inhibition sharpens PN responses to sparse stimuli. To investigate the role of GABA_A_ and GABA_B_ receptors on the temporal sharpening of presynaptic voltage responses, we first conducted pharmacological manipulations and measured voltage responses directly in the presynaptic terminals. While pharmacological inhibition of GABA_B_ receptors (CGP 100 µm) shows no effect on presynaptic voltage responses to a 1:5 Eb pulse (Fig. 4*A*), the inhibition of GABA_A_ receptors (PTX 200 µm) appears to increase response magnitude (Fig. 4*B*). An explanation for the difference with previous studies demonstrating that CGP54626 increases neuronal activity in presynaptic terminals (Olsen and Wilson, 2008; Root et al., 2008) could be that the pharmacological inhibition of GABA_B_ receptors might have a larger effect on presynaptic Ca^2+^ and synaptic transmission than on presynaptic voltage. Interestingly, simultaneous pharmacological inhibition of GABA_A_ and GABA_B_ receptors induces prolonged presynaptic voltage responses to 1:5 and 1:3 Eb pulses (Fig. 4*C*,*E*), while odor kinetics are unaltered between the measurements (Fig. 4*D*,*F*). Quantification of peak response magnitude and sharpness indicates that only the simultaneous pharmacological inhibition of GABA_A_ and GABA_B_ receptors significantly increases amplitude (Fig. 4*G*) and reduces sharpness of OSN presynaptic voltage responses (Fig. 4*H*), which also demonstrates the effectiveness of both CGP54626 and PTX. This is consistent with the previous finding that disinhibition in the AL is poorly achieved by either CGP54626 or PTX alone but is fully achieved by the simultaneous application of these drugs (Olsen and Wilson, 2008). In general, the conclusion of this experiment is consistent with the finding that presynaptic inhibition in OSN terminals is mediated by GABA_A_ and GABA_B_ receptors (Olsen and Wilson, 2008). The more severe disruption of presynaptic sharpening for 1:3 Eb pulses than for 1:5 (Fig. 4*H*) is consistent with the concentration-dependent sharpness of peripheral responses (Fig. 2*F*). Sustained activity after the blocking of presynaptic inhibition is higher than one would expect based on the sustained peripheral firing rates (Fig. 1). This might be because low-frequency firing is better reflected by ArcLight in comparison to high-frequency firing. It could also be that it is not only spiking activity but also slow changes in membrane potential that travel to the presynaptic terminals and underlie presynaptic inhibition. This hypothesis is supported by the substantially prolonged Ca^2+^ kinetics we observe in the presynaptic terminals. Our pharmacological studies suggest that both GABA_A_ and GABA_B_ receptors mediate the presynaptic inhibition of OSNs to implement temporal contrast enhancement of sustained peripheral responses. To test whether the combined activity of GABA_A_ and GABA_B_ receptors can mediate temporal contrast enhancement in other glomeruli, we measured presynaptic voltage responses of OR42a-expressing OSNs, which reside in the maxillary palps and whose terminals project to the VM7 glomerulus (Fig. 4*I*). For VM7, lateral inhibition can be reduced by removal of the antennae which increases odor responses in VM7 PNs (Olsen and Wilson, 2008). This disinhibition can be mimicked only by simultaneous blockage of GABA_A_ and GABA_B_ receptors (Olsen and Wilson, 2008), suggesting that both receptors are present at the presynaptic terminals of OR42a-expressing OSNs. We find that removal of the antennae increases presynaptic voltage responses to 1:5 Eb and also reduces presynaptic sharpening (Fig. 4*I*). This suggests that the implementation of temporal contrast enhancement via the combined activity of presynaptic GABA_A_ and GABA_B_ receptors is a general phenomenon in the *Drosophila* AL. However, our findings do not rule out the possibility that GABA_A_- and/or GABA_B_-mediated inhibition of other neurons in the AL olfactory network are involved.

**Figure 5.**
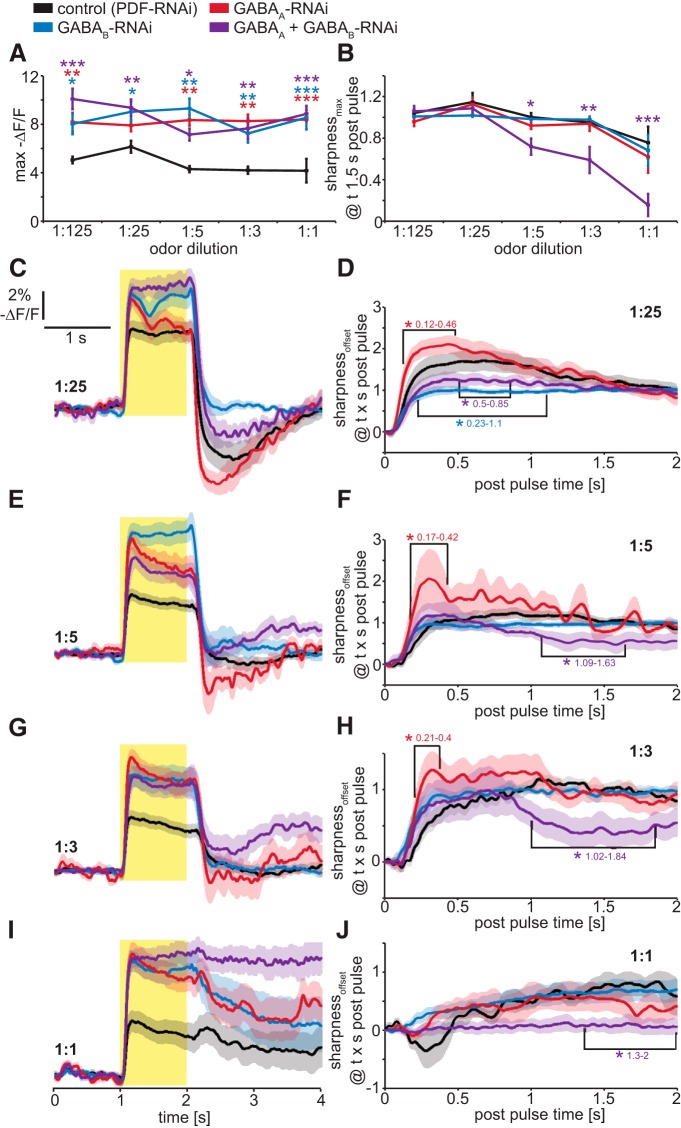
Temporal contrast enhancement in OSN presynaptic terminals is mediated by presynaptic GABA_A_ and GABA_B_ receptors as demonstrated by cell-specific RNAi-mediated knockdown. ***A***, Maximum presynaptic voltage responses are increased by individual and simultaneous RNAi-mediated knockdown of GABA_A_ (8-10G) and GABA_B_ receptors. Mean ± SEM; *n* = 8. Statistical analysis: two-way repeated-measures ANOVA with Bonferroni *post hoc* test (asterisks are color coded to indicate pairwise comparisons vs control), *p < 0.05; **p < 0.01; ***p < 0.001. ***B***, Temporal contrast enhancement of DM2 presynaptic voltage responses is unaffected by RNAi-mediated knockdown of either GABA_A_ (8-10G) or GABA_B_ receptors individually in OR22a-expressing OSNs. Simultaneous knockdown of GABA_A_ (8-10G) and GABA_B_ receptors reduces temporal contrast enhancement at odor intensities of 1:5 and higher. Mean ± SEM; *n* = 8. Statistical analysis: two-way repeated-measures ANOVA with Bonferroni *post hoc* test, *p < 0.05; **p < 0.01; ***p < 0.001. ***C***, ***E***, ***G***, ***I***, Presynaptic DM2 voltage responses to 1 s Eb pulses of gas-phase dilutions 1:25 (***C***), 1:5 (***E***), 1:3 (***G***), and 1:1 (***I***) in flies expressing either, or both, GABA_A_ (8-10G) and GABA_B_-RNAi in OR22a-expressing OSNs. Mean ± SEM; *n* = 8-10. ***D***, ***F***, ***H***, ***J***, Time-dependent sharpness coefficient to analyze the time window in which GABA receptor knockdown affects contrast enhancement. After a 1:25 pulse (***D***) knockdown of GABA_B_ receptors and simultaneous knockdown of GABA_A_ (8-10G) and GABA_B_ receptors affect sharpness during the hyperpolarization phase immediately after odor offset. Knockdown of GABA_A_ receptors leads an increase in postpulse hyperpolarization for 1:25 (***D***), 1:5 (***F***), and 1:3 (***H***). Contrast enhancement of sustained activity later than 1 s after the odor offset is only achieved by simultaneous knockdown of GABA_A_ and GABA_B_ receptors in OR22a-expressing OSNs, indicating a combined role for these receptors. Statistical analysis: two-way repeated-measures ANOVA with Bonferroni *post hoc* test, *p < 0.05.

To address this issue, we genetically suppressed GABA receptor expression in OSNs innervating a single glomerulus by expressing GABA receptor-directed RNAi hairpin constructs using *OR-GAL4* drivers, and measured the effects on odor-induced presynaptic electrical responses of OR22a-expressing OSNs (Fig. 5). As a control for possible nonspecific RNAi effects, we expressed RNAi directed against the neuropeptide PDF, which is not expressed in OSNs. To achieve RNAi-mediated knockdown of GABA_A_ or GABA_B_ receptors, we individually expressed either GABA_A_-RNAi (8-10G; Liu et al., 2007, 2009) or GABA_B_-R2-RNAi (Root et al., 2008), respectively, in OR22a-expressing OSNs. Each of these RNAi lines has previously been established to effectively downregulate their corresponding GABA receptor subtypes (Liu et al., 2007, 2009; Root et al., 2008). Individual and simultaneous knockdown of GABA_A_ or GABA_B_ receptors increases the peak magnitude of presynaptic voltage responses (Fig. 5*A*). Consistent with our pharmacological results, only simultaneous knockdown of GABA_A_ and GABA_B_ receptors reduces the sharpness of presynaptic voltage responses (Fig. 5*B*). To investigate at what time after the odor offset knockdown of GABA receptors affect presynaptic contrast enhancement, we performed a time-dependent analysis of the sharpness coefficient (sharpness_offset_; Fig. 5*C–J*). Interestingly, knockdown of GABA_A_ receptors increases sharpness during the immediate postpulse hyperpolarization phase (Fig. 5*D*,*F*,*H*). This could be a result of increased GABA_B_ receptor activity, which could be due to homeostatic processes triggered by the downregulation of GABA_A_ receptors. This hypothesis is supported by the finding that knockdown of GABA_B_ receptors, and the simultaneous knockdown of GABA_A_ and GABA_B_ receptors, significantly reduce sharpness during the immediate postpulse hyperpolarization phase (Fig. 5*D*). Sharpness of sustained neuronal activity, which occurs only at higher intensities, is reduced only by the simultaneous knockdown of GABA_A_ and GABA_B_ receptors (Fig. 5*F*,*H*,*J*). For 1:5 and 1:3 dilutions, sharpness is reduced during a very narrow time window of 1.02 and 1.84 s after the odor offset (Fig. 5*F*,*H*). For 1:1 dilution, the already prolonged neuronal activity in the control is further increased with simultaneous knockdown of GABA_A_ or GABA_B_ receptors and leads to reduced sharpness 1.3 s after odor offset (Fig. 5*I*,*J*). These cell-specific genetic manipulations establish that the activity of both GABA_A_ and GABA_B_ receptors expressed by the OSNs innervating a single glomerulus increase the temporal contrast of presynaptic responses to high-intensity odor stimuli.

### Presynaptic inhibition of OSNs accelerates behavioral responses to odor offset

**Figure 6.**
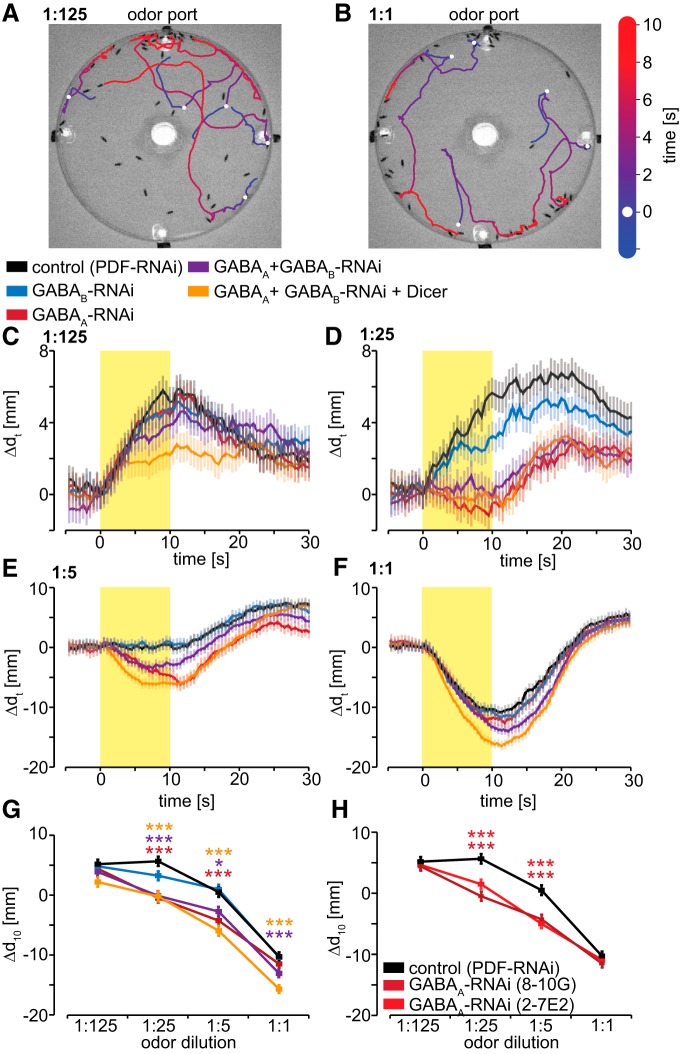
Presynaptic OSN GABA receptors affect innate olfactory attraction and avoidance. ***A***, ***B***, Representative trajectories of control flies (PDF-RNAi) in the olfactory arena. The white dot indicates the position of the fly at the beginning of the 10 s odor pulse. The odor enters the arena from the top odor port. Trajectories indicate movement toward the odor port during a 10 s 1:125 Eb pulse (***A***), and away from the odor port during a 1:1 Eb pulse (***B***). ***C***, ***D***, Behavioral responses to 10 s 1:125 (***C***) and 1:25 (***D***) Eb pulses. We defined Δ*d_t_* as the difference in distance from the odor port between odor onset and time *t* (Δ*d_t_* = *d*_0_ − *d_t_*), such that positive Δ*d_t_* values reflect movement toward the odor port (i.e, attraction) and negative values reflect movement away (i.e., avoidance). Control flies (PDF-RNAi) are attracted to the odor port during Eb pulses of these intensities, and this attraction is inhibited by RNAi-mediated knockdown in OR22a-expressing OSNs of GABA_A_ receptors individually or GABA_A_ and GABA_B_ receptors simultaneously. Mean ± SEM; ***E***, ***F***, Control flies (PDF-RNAi) avoid the odor port during 10 s at 1:5 (***C***) and at 1:1 (***D***) Eb pulses, and this avoidance is increased by RNAi-mediated knockdown in OR22a-expressing OSNs of GABA_A_ receptors individually or GABA_A_ and GABA_B_ receptors simultaneously. Mean ± SEM; ***G***, Net distance moved at the end of 10 s Eb pulses of the indicated gas-phase dilutions. For Eb dilutions of 1:25 or 1:5, knockdown in OR22a-expressing OSNs of GABA_A_ receptors individually or simultaneous knockdown of GABA_A_ and GABA_B_ receptors, increases avoidance of the odor port. For Eb dilution of 1:1, only simultaneous knockdown of GABA_A_ and GABA_B_ receptors increases avoidance. Mean ± SEM, *n* = 650–800 total flies per genotype and concentration assayed in at least 10 independent experiments. Statistical analysis: two-way ANOVA followed with Bonferroni *post hoc* test (asterisks are color coded to indicate pairwise comparisons vs control), *p < 0.05; ***p < 0.001. ***H***, Expression of either of two different GABA_A_-RNAi transgenes in OR22a-expressing OSNs increases avoidance. Mean ± SEM. Statistical analysis: two-way ANOVA with Bonferroni *post hoc* test, ***p < 0.001.

We next sought to determine whether presynaptic inhibition of OSNs influences the time course of innate behavioral responses to time-varying olfactory stimuli. We used automated fly tracking software adapted from open-source code (see Materials and Methods) to track the locomotor responses of walking flies to Eb pulses delivered from an odor port into a circular arena containing 30–50 flies. To properly compare the behavioral experiments with the physiological experiments in Figure 5, we used the same control expressing PDF-RNAi (without ArcLight). This is advantageous over using inbred parental lines as inbreeding can affect locomotor activity (Manenti et al., 2015). Although we use the same odor dilutions as in the physiological experiments, the odor intensities that the flies experience could be quite different due to distance from the odor port and the fact that the behavioral arena is closed. To quantify attractive or aversive responses, we calculated the distance of each fly from the odor port over time, relative to its initial position at the beginning of each trial. Ten second pulses of 1:125 Eb induce attraction, with flies moving closer to the odor port (Fig. 6*A*). In contrast, 10 s pulses of 1:1 Eb induce avoidance, with flies moving away from the odor port (Fig. 6*B*). This is consistent with the previous observation that low concentrations of Eb solely activate OSNs mediating innate attraction, while higher concentrations recruit additional OSNs mediating innate avoidance (Semmelhack and Wang, 2009). Control flies expressing PDF-RNAi in OR22a-expressing OSNs innervating DM2 are attracted to pulses of 1:125 and 1:25 Eb (Fig. 6*C*,*D*). Flies expressing GABA_A_-RNAi (8-10G) alone or simultaneously expressing GABA_A_-RNAi (8-10G) and GABA_B_-RNAi exhibit reduced attraction (Fig. 6*C*,*D*). Control flies are neither attracted nor repelled by pulses of 1:5 Eb (Fig. 6*E*), consistent with the interpretation that this intensity of Eb stimulates OSNs mediating attraction and avoidance to a relative extent that counterbalances behavioral responses. In stark contrast to control flies, GABA_A_-RNAi (8-10G) and GABA_A_-RNAi (8-10G) + GABA_B_-RNAi flies are repelled by 1:5 Eb pulses (Fig. 6*E*). All flies, regardless of genotype, are strongly repelled by pulses of 1:1 Eb, with increased avoidance in GABA_A_-RNAi (8-10G) + GABA_B_-RNAi flies (Fig. 6*F*). Avoidance of 1:5 and 1:1 dilutions is even stronger in flies expressing Dicer together with GABA_A_-RNAi (8-10G) + GABA_B_-RNAi, which underlines the specificity of the behavioral changes. Statistical analysis supporting these conclusions is shown in Figure 6*G*. The expression of a different GABA_A_-RNAi (2-7E2; Liu et al., 2009) in OR22a-expressing OSNs also increases avoidance (Fig. 6*H*), supporting the specificity of these GABA_A_-RNAi effects. In light of the study by Semmelhack and Wang (2009), who did not observe any changes in valence after silencing DM2, our findings of increased presynaptic electrical activity in DM2 leading to increased avoidance could be explained by altered network activity. Increased activity in DM2 mediated by the knockdown of GABA_A_ receptors (Fig. 5) could, for example, increase lateral inhibition, which could affect other glomeruli, leading to increased avoidance.

**Figure 7.**
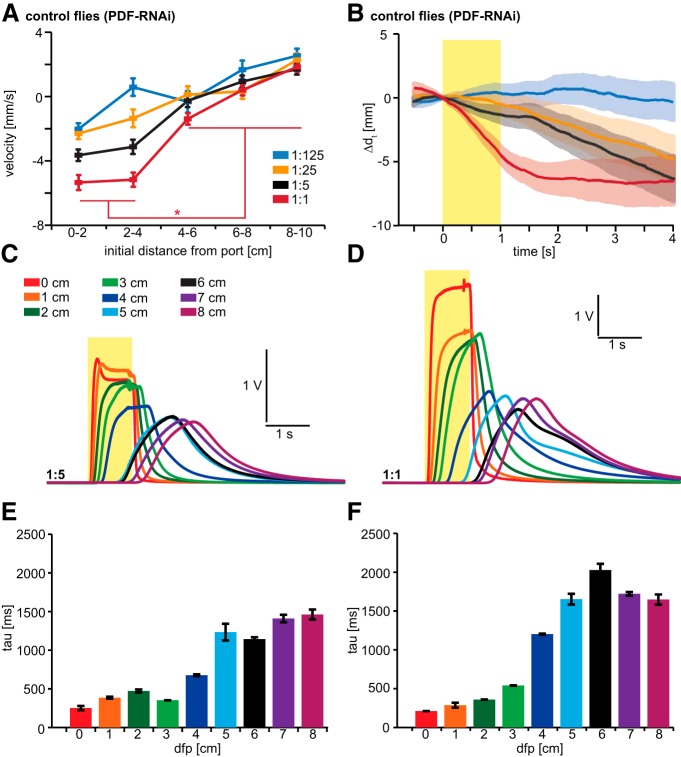
Behavioral responses to quick 1 s Eb pulses are dependent on temporal odor dynamics in the behavioral chamber. ***A***, Velocity of flies during and after 1 s Eb pulses is dependent on initial position within the olfactory arena at initiation of the odor pulse. Control flies expressing PDF-RNAi in OR22a-expressing neurons exhibit strong avoidance only to 1:1 Eb and only when initial position in the arena is ≤4 cm from the odor port. Mean ± SEM; *n* = 116–172 total flies per genotype and concentration assayed in at least 10 independent experiments. Statistical analysis: two-way ANOVA with Bonferroni *post hoc* test, *p < 0.05. ***B***, Behavioral responses of control flies (initial position to port <5 cm) to 1 s Eb pulses of gas-phase dilutions, as indicated in ***A***. Mean ± SEM. ***C***, ***D***, Mean PID recordings (*n* = 5) in the behavioral chamber showing odor dynamics of 1 s odor pulses of 1:5 (***C***) and 1:1 (***D***) that vary dependent on the distance to the odor port. ***E***, ***F***, Tau represents the time that the odor stimulus takes to reach 36.8% of its peak value. For 1:5 (***E***) and 1:1 (***F***), tau drastically increases after a distance of 3–4 cm from the odor port, indicating that flies within 3 cm from the odor port experience a fast increase and decrease in odor intensity, while flies that are >3 cm away from the odor port experience more gradual changes in odor intensity. Mean ± SEM. *n* = 5.

**Figure 8.**
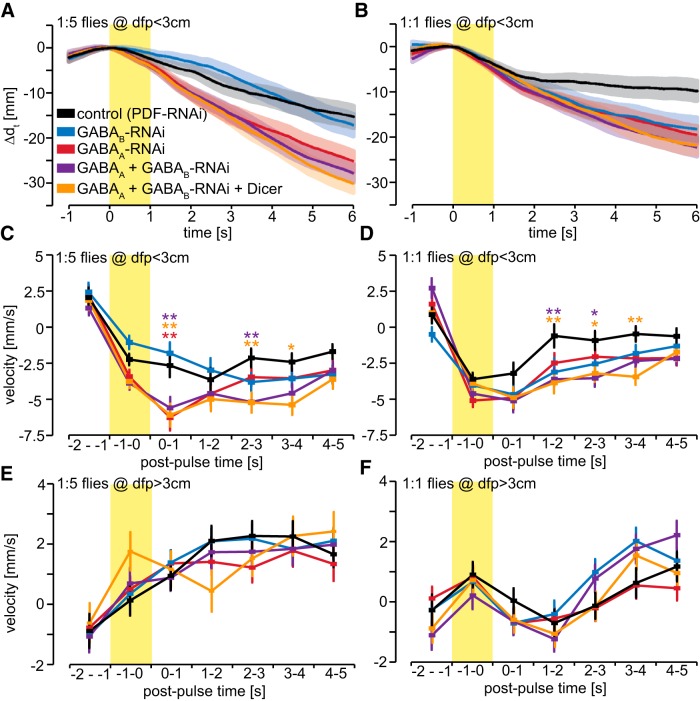
Presynaptic OSN GABA receptors accelerate behavioral responses to odor pulse termination. ***A***, ***B***, Behavioral responses of flies within 3 cm from the odor port show avoidance during and after a 1 s Eb pulse of 1:5 (***A***) and 1:1 (***B***). Mean ± SEM; *n* = 100–200 total flies per genotype and concentration assayed over at least 10 independent experiments. ***C***, Average velocity of flies within 3 cm from the odor port during and after a 1 s 1:5 Eb pulse. During and immediately after the odor pulse, the velocity away from the odor port is significantly increased by individual knockdown of GABA_A_ receptors and simultaneous knockdown of GABA_A_ and GABA_B_ receptors. The velocity between 2 and 4 s after the odor pulse is significantly increased by simultaneous knockdown of GABA_A_ and GABA_B_ receptors. Statistical analysis: two-way ANOVA with Bonferroni *post hoc* test (asterisks are color coded to indicate pairwise comparisons vs control), *p < 0.05; **p < 0.01. ***D***, Average velocity during 1:1 Eb odor pulses is unaffected by the knockdown of GABA receptors. However, simultaneous knockdown of GABA_A_ and GABA_B_ receptors significantly prolongs avoidance between 1 and 4 s after termination of the odor pulse. Statistical analysis: two-way ANOVA with Bonferroni *post hoc* test, *p < 0.05; **p < 0.01. ***E***, ***F***, Average velocity of flies that are >3 cm away from the odor port is unaffected by knockdown of GABA receptors for 1:5 (***E***) 1:1 (***F***) Eb odor pulses. Mean ± SEM. *N* = 200–300 flies.

These sustained attractive and aversive responses to 10 s odor pulses likely reflect chemotactic responses to steady-state odor gradients in the olfactory arena. To probe the effects of presynaptic OSN inhibition on the perception of a time-varying stimulus, we delivered 1 s odor pulses and focused our attention on behavioral responses during and after termination of the pulse (Figs. 7, 8). Responses of individual flies to brief odor pulses depend heavily on odor dilution and the initial distance from the odor port at the time of odor onset (Fig. 7*A*,*B*). Flies that are initially close to the odor port avoid it, while flies that are initially far away do not respond. We focused on the high odor intensities and used the PID to measure odor dynamics within the behavioral arena (Fig. 7*C*,*D*). Interestingly, within 3 cm of the odor port flies experience odor dynamics in a way that is similar to entering and exiting of a plume, with a rapid increase of odor intensity and a fast decline. More than 3 cm away from the odor port, the changes in odor intensity are more gradual. To visualize this, we calculated tau, which represents the time taken from the peak PID value to 36.8% of the PID value (Fig. 7*E*,*F*). To study the behavior of flies upon exiting plume-like odor dynamics, we focused on those flies whose initial position was within 3 cm from the odor port (Fig. 8*A–D*).

Immediately after a 1 s odor pulse (0–1 s postpulse time) of 1:5 Eb GABA_A_-RNAi (8-10G) or GABA_A_-RNAi (8-10G) plus GABA_B_-RNAi (plus Dicer) in OR22a-expressing OSNs, innervating DM2 increases velocity away from the odor port (Fig. 8*A*,*C*). This likely reflects increased avoidance, which was also shown for the 10 s odor pulse (Fig. 6*G*). The velocity between 2 and 4 s after termination of the odor pulse remains significantly higher only in flies expressing GABA_A_-RNAi (8-10G) and GABA_B_-RNAi flies (Fig. 8*C*). Control flies exhibit stronger avoidance response to pulses of 1:1 Eb than to those of 1:5 Eb, and, interestingly, this avoidance terminates rapidly upon termination of the pulse (Fig. 8*B*,*D*). As the neuronal activity of DM2 presynaptic terminals to 1:1 odor pulses would suggest an even more prolonged avoidance, this could demonstrate how the activity of multiple glomeruli is used to balance and switch innate behavior between attraction and avoidance. However, while GABA receptor knockdown does not affect avoidance during or immediately after the 1:1 Eb pulse, simultaneous knockdown of GABA_A_ and GABA_B_ receptors prolongs avoidance between 1 and 4 s after termination of the odor pulse (Fig. 8*D*). In contrast, GABA receptor knockdown did not affect flies that are >3 cm away from the odor port and experience a more gradual change in odor dynamics (Fig. 8*E*,*F*). This suggests that the combined activity of presynaptic GABA_A_ and GABA_B_ receptors, which mediates gain control and temporal contrast enhancement in olfactory sensory neurons, enhances detection of the termination of the odor pulse.

## Discussion

**Figure 9.**
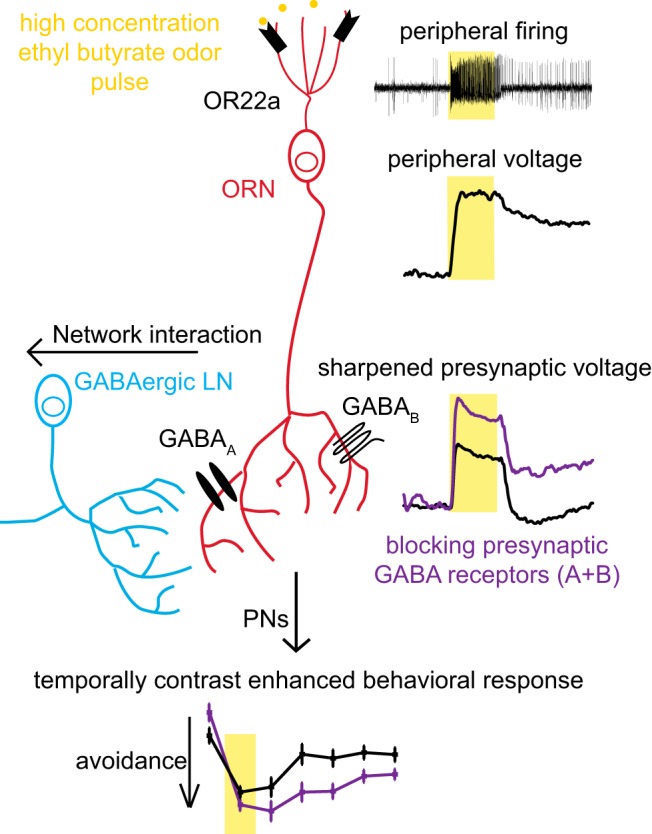
GABA_A_ and GABA_B_ receptors mediate presynaptic inhibition of OSNs to implement temporal contrast enhancement of sustained peripheral responses. A 1 s ethyl butyrate odor pulse (yellow boxes) of high concentration induces sustained peripheral neuronal activity in dendrites and cell bodies of OR22a-expressing OSNs. GABAergic LNs activate GABA_A_ and GABA_B_ receptors, leading to temporally sharpened odor responses in presynaptic terminals. Presynaptic sharpening contributes in mediating temporal contrast enhancement improving the detection of termination of a high-intensity odor pulse.

Lateral inhibition in the visual system improves environmental perception by enhancing contrast vision to enable accurate spatial edge detection (Kuffler, 1953; Buldyrev and Taylor, 2013). Temporal edge detection in the auditory system improves sound localization (Chait et al., 2008). Here we address whether similar mechanisms exist in the olfactory system that improve odor edge detection (Fig. 9, summary). We find that high-intensity odor pulses induce sustained peripheral responses in OSNs (Figs. 1, 2, 9). We use optical electrophysiology to visualize that sustained peripheral responses undergo contrast enhancement by presynaptic GABA receptors to generate sharper responses in OSN presynaptic axon terminals in the AL (Figs. 4, 5, 9). Furthermore, the combined activity of presynaptic GABA_A_ and GABA_B_ receptors modulates the kinetics of innate olfactory behavior after termination of an odor pulse (Figs. 8, 9).

Our voltage and Ca^2+^ measurements reveal contrast enhancement of presynaptic OSN electrical activity but not presynaptic intracellular Ca^2+^ (Fig. 3). It is possible that high odor intensities induce Ca^2+^ release from internal stores, resulting in sustained presynaptic Ca^2+^ increases (Murmu et al., 2010, 2011). Alternatively, sustained peripheral responses could activate voltage-gated Ca^2+^ channels along the axons of the OSNs (Murmu et al., 2010), with sustained presynaptic Ca^2+^ increases mirroring sustained peripheral responses. As electrical recordings from single PNs indicate that membrane depolarization is tightly coupled to neurotransmitter release (Nagel et al., 2015), it is likely that sharpened presynaptic voltage responses are faithfully propagated even when presynaptic intracellular Ca^2+^ remains high. This could be because Ca^2+^ indicators report bulk cytoplasmic Ca^2+^ in the nanomolar to micromolar range, and not the substantially higher Ca^2+^ transients in the Ca^2+^ channel-associated microdomains that drive synaptic vesicle release (Llinás et al., 1992; Oheim et al., 2006; Matkovic et al., 2013). While bulk cytoplasmic Ca^2+^ levels could remain elevated after a train of action potentials invade the presynaptic terminals, the microdomain concentration at synaptic release sites may have already declined below the threshold for triggering release. Consistent with this interpretation of our observations, odor stimuli eliciting sustained Ca^2+^ increases in OSN presynaptic terminals induce substantially more abbreviated Ca^2+^ increases in postsynaptic PN dendrites (Asahina et al., 2009). However, it is also possible that postsynaptic inhibition contributes to temporal sharpening of PN responses (Wilson and Laurent, 2005; Fujiwara et al., 2014).

Lateral inhibition in the AL has extensively been studied in different insect species. In locust, GABAergic local interneurons have been shown to synchronize oscillations between odor-coding neural assemblies in the AL (MacLeod and Laurent, 1996). In the honey bee, these synchronized oscillations have been shown to be essential for the discrimination of molecularly similar odorants (Stopfer et al., 1997). Moreover, calcium imaging studies in the honey bee have demonstrated that local interneurons mediate global inhibition in the AL to enhance spatial contrast between glomeruli (Sachse and Galizia, 2002). In the hawkmoth, *Manduca sexta*, blocking inhibition in the AL impairs the localization of odor sources by affecting the temporal firing pattern in PNs (Lei et al., 2009). In *Drosophila* it has become evident that presynaptic GABA receptors play a crucial role in mediating lateral inhibition in the AL (Olsen and Wilson, 2008; Root et al., 2008). While presynaptic GABA receptors have been shown to be essential for odor object localization (Root et al., 2008), we show that presynaptic GABA receptors also enhance temporal contrast within glomeruli to improve the detection of the temporal structures of odor plumes.

*Drosophila melanogaster* live, feed, and reproduce on fermenting fruits. To locate fermenting fruit, *Drosophila* navigate via plumes of odors (Gaudry et al., 2012; van Breugel and Dickinson, 2014). Gain control mediated by presynaptic inhibition is an important mechanism for maintaining sensitivity to a wide range of experienced odor intensities (Olsen and Wilson, 2008; Root et al., 2008). While GABA_B_-mediated presynaptic gain control is known to be important for localizing pheromone-emitting objects (Root et al., 2008), no behavioral role for presynaptic GABA_A_ receptors has previously been reported. Here we show that presynaptic OSN GABA_A_ receptors modulate innate behavioral responses to the fruit-related odor Ethyl butyrate (Eb) (Figs. 6, 8). While blocking synaptic output in DM2 was previously reported to have no behavioral consequence (Semmelhack and Wang, 2009), our RNAi-mediated GABA_A_ knockdown results indicate that increased neuronal activity in DM2 leads to increased avoidance (Figs. 6, 8). A possible explanation could be that increased activity in DM2 alters network activity in the AL affecting other glomeruli that mediate attraction or aversion. Interestingly, RNAi-mediated presynaptic knockdown of GABA_B_ receptors individually had no statistically distinguishable effect on behavioral responses to Eb (Figs. 6, 8). While GABA_B_ receptors play an important role in sustained pheromone-related behaviors and are differentially expressed across glomeruli (Root et al., 2008), GABA_A_ receptors might be more important for processing transient fruit-related odor stimuli. However, future immunohistological studies need to show the presence of presynaptic GABA_A_ receptors and their distribution across different glomeruli. It could also be that GABA_B_ receptors affect the AL network activity in a different way than GABA_A_ receptors. At the highest odor intensity tested, only simultaneous knockdown of GABA_A_ and GABA_B_ receptors in DM2 increased aversive behavior (Fig. 6*F*,*G*). This demonstrates a combined role for GABA_A_ and GABA_B_ receptors in mediating presynaptic inhibition as has previously been observed in physiological studies (Olsen and Wilson, 2008). Moreover, we also visualize the combined role for GABA_A_ and GABA_B_ receptors in mediating temporal contrast enhancement of presynaptic electrical responses. For the first time, we link presynaptic inhibition mediating gain control and temporal contrast enhancement (Figs. 4, 5) to behavioral responses after odor pulse termination (Fig. 8). This suggests that the combined participation of GABA_A_ and GABA_B_ receptors could be an advantage for animals that encounter a very wide dynamic range of odor stimuli.

Temporal sharpening of olfactory information could also play an important role in associative learning. During associative learning in mammals and insects, temporally limited olfactory stimuli determine a critical time window for the integration of other sensory information, such as sugar as a reward or electric shock as a punishment (Tully and Quinn, 1985; Hammer and Menzel, 1995; Delamater et al., 2014). In single neurons implicated in learning, GABAergic inhibition has been shown to truncate neuronal activity and thus has been hypothesized to define the time window for coincidence detection (Pouille and Scanziani, 2001; Mittmann et al., 2005; Raccuglia and Mueller, 2014). In fact, altering the degree of GABAergic inhibition or artificially activating GABA receptors during learning interferes with the formation of associative memories in insects (Liu et al., 2007; Liu and Davis, 2009; Raccuglia and Mueller, 2013). The GABAergic presynaptic temporal contrast enhancement we reveal here could play a role in determining a concentration-invariant critical time window for enhancing the temporal accuracy of associative memories.

How could presynaptic inhibition underlie temporal contrast enhancement? Presynaptic and postsynaptic inhibition in the AL are mediated by multiple GABAergic LNs, which receive excitatory odor-induced inputs from multiple glomeruli and then inhibit OSN presynaptic terminals and PN postsynaptic dendrites (Stocker et al., 1990; Ng et al., 2002). Most individual LNs innervate a large number of glomeruli (Chou et al., 2010; Seki et al., 2010). OSN presynaptic activity could be modulated by the sustained activity of LNs (Wilson et al., 2004; Wilson and Laurent, 2005; Chou et al., 2010; Nagel et al., 2015), or a transient increase in LN activity upon a sharp decline in odor intensity (Nagel et al., 2015). A recent study has shown the presence of GABAergic LNs, which respond to odor offsets (Nagel and Wilson, 2016) and would be perfectly suited to mediate temporal contrast enhancement. It is also possible that slow kinetics of metabotropic GABA_B_ receptors mediate sustained inhibition after a rapid decline in odor intensity (Wilson and Laurent, 2005). Given the combined role of GABA_A_ and GABA_B_ receptors in temporal contrast enhancement, it is likely that a combination of the intrinsic physiological properties of LNs and the kinetics of GABA receptor activation contribute to shaping the time course of presynaptic inhibition that underlies temporal contrast enhancement. Future studies are required to probe the role of this mechanism in actual plume-guided navigation.

## Synthesis

The decision was a result of the Reviewing Editor Muriel Thoby-Brisson and the peer reviewers coming together and discussing their recommendations until a consensus was reached. A fact-based synthesis statement explaining their decision and outlining what is needed to prepare a revision is listed below. The following reviewer(s) agreed to reveal their identity: Jean-René Martin

Dear authors,

Please find below the comments made by the two reviewers regarding your manuscript.

General comments:

The manuscript describes the presynaptic temporal contrast enhancement in olfactory sensory neurons (OSNs), in Drosophila. While the contrast enhancement mediated by lateral inhibition within the nervous system has already been described for visual and auditory stimuli, up to now, it was not yet clearly demonstrated for the olfactory system. Here, the authors use a combination of sophisticated physiological approaches, as the classical electrophysiology (single sensillum recording - SSR), the optical recording of electrical activity (ArcLight), and the optical calcium imaging (GCaMP6f), to demonstrate that the sustained peripheral responses are temporally sharpened. In a step further, they use the pharmacological approaches corroborated by the genetic tools of Drosophila as the targeted expression of RNAi to knock-down specifically the GABA receptors within the OSNs to demonstrate that these temporal responses are sharpened by the combined activity of the two types of inhibitory GABA receptors (GABA-A and GABA-B) to generate a contrast-enhanced voltage responses in OSN axon terminals. In addition, to demonstrate a behavioral significance of these physiological mechanisms, they developed an elegant method to precisely measure the avoidance and the attraction to the odors. In summary, for the first time, they link the presynaptic inhibition mediating gain control and temporal contrast enhancement to behavioral response after odor pulse termination.

The paper is well written and well documented. However the reviewers raised several major and minor comments (detailed below) that have to be addressed in order to improve the clarity and readability of the current MS.

Major comments:

-While the authors have performed a broad series of experiments at the functional and behavioral level, the broad conclusion seems to fall a bit short considering that the authors examined only a single OR-type using a single odor. The results could be seen in a broader context if a few representative experiments would be added showing that blocking and silencing of both GABA receptor in other OR-type expressing sensory neurons leads to the same results. We therefore encourage the authors to select at least one other OR-type to show that the functional effect is a general phenomenon. Moreover, GABAergic inhibition at the level of the antennal lobe has been intensively studied in other insect species such as moths, honeybees and locusts long before it has been examined in the fruit fly. These studies should be mentioned and discussed in the light of these new finding in the discussion section.

- Regarding the precise localization of the GABA-receptors in the antennal lobes, though the GABA-B receptors have been well demonstrated to be localized to the axon terminals of the OSNs (by immunohistochemistry), the localization of the GABA-A receptors remain more elusive (as far as we know, only physiological approaches suggest that). This point should be mentioned in the manuscript.

-Although the behavioral experiments are elegantly and well conducted, in few places, the differences are obviously quite subtle. Considering that in several other behavioral experiments, as the learning and memory, locomotor activity or else, it is generally mandatory to perfectly control the genetic background of all the fly lines used (in other words: homogenize the genetic background or "cantonise" the lines), we remark that for the olfactory system, these strictness and constraints are generally not required. Nevertheless, the authors should keep in mind this rigor and thus remain careful in their general interpretation.

- Page 12 / Figure 4: What is the author's explanation regarding that only both drugs, PTX and CGP, induce a significant effect? Why isn't any washout shown? They washout would verify that the increase in response is indeed due to drug application. The concentrations used for both GABA blockers (PTX: 200 µM / CGP: 100 µM) are unusual high. Other studies using these blockers in Drosophila have used concentrations of 5 µM for PTX and 50 µM for CGP. Why did the authors use such high concentration? Did the author apply lower concentrations as well? Please add the drug concentrations also to the Figure legend of Figure 4.

- Page 13 / Figure 5: Please add the exact genotype of the control line used! Why didn't the authors test both parental lines as controls? That would improve considerably the significance of the effects shown!

- Some statistics and quantifications should be added. For instance: Page 8 / Figure 1D,E: The authors state that ArcLight expression does not alters the excitability of sensory neurons. However, this statement needs a statistical verification. And on Page 10: Please explain in more detail how you quantified the temporal contrast enhancement and what the value actually means. Has this parameter been used in other studies before? Why did you choose this measure?

- General comments for all figures: for clarity we suggest to add axes for each graph, in particular the time courses of the odor responses. Moreover, please add for each graph the odor stimulation as a shaded area or a bar.

- In order to represent all results in a comprehensive and clear way, we suggest adding a network scheme in the end that summarizes all findings of this MS.

Minor comments:

- The title is very long and the authors should do an effort to shorten it.

- The authors use throughout the MS the expression 'the lower dilution of ...'. This expression is a bit confusing and we suggest changing it to 'at a higher concentration (1:5 dilution)'

- line 146: in Materials & Method: Why did the authors perform the behavioral experiences at 30{degree sign}C? This is not common, since 30{degree sign}C starts to induce heat-shock proteins. The authors should justify.

- line 214: that that (remove one "that")

- line 250: should be 1:10 instead of 1:3 (according to Fig. 3I)

- Page 11, line 253: the following argument is difficult to understand: 'At 1:1 it might be harder to compensate the sustained peripheral activity making it harder to detect this temporal sharpening'

- line 307 : "and" instead of "or"

- Page 13, line 313: Please clarify the following argument: 'This could be due to compensatory effects mediated by GABAB receptors.'

- line 315: "and" instead of "or"

- line 318 : "and" instead of "or"

- Page 13, line 324: How do the authors know that the sharpness of the odor-evoked responses reflects indeed contrast enhancement?

- Page 14, line 347: Replace 'stark' by 'strong'.

- line 364: why 7A+ (remove + ?)

- line 478: please complete the reference (page number ..., etc.)

- line 535: "2015" and not "2014" and please complete the reference (Vol, page number, etc.)

- line 653: G instead of H

- line 658: H instead of I

- Page 14 / Figure 6: Have the flies been sorted according to their gender? If so, did the authors observe any difference between males and females?

- We suggest condensing Table 1 in a way that it fits in a single page.
